# Identification of E2F/DP gene family and their expression analysis under cold stress in rice

**DOI:** 10.3389/fpls.2026.1750382

**Published:** 2026-02-16

**Authors:** Yaojia Ma, Shujing Mu, Jun Zhao, Jiarui Han, Mo Zhu, Qi Wang, Mojun Chen, Rihua Piao, Fanmei Meng, Xin Wang, Changhong Xu, Hongguang Ju, Yongfeng Yan, Xiangguo Liu

**Affiliations:** 1 College of Agriculture, Yanbian University, Yanji, China; 2 Institute of Rice Research/Institute of Agricultural Biotechnology, Jilin Academy of Agricultural Sciences (Northeast Innovation Center of Agricultural Science and Technology in China), Changchun, China; 3 Jilin Provincial Key Laboratory of Agricultural Biotechnology, Changchun, China

**Keywords:** bioinformatics, cold stress, E2F/DP gene family, phylogenetic analysis, rice (Oryza sativa L.)

## Abstract

The rice E2F/DP gene family plays a crucial role in regulating the cell cycle and responses to environmental stresses. In this study, we identified and analyzed eight members of the rice E2F/DP gene family (*OsE2F1* to *OsE2F8*) using the RiceSuperPIRdb database. Each protein contains the conserved E2F_TDP domain. Phylogenetic analysis classified the E2F/DP proteins into three groups. Physicochemical property analysis revealed that the E2F/DP proteins are hydrophilic, with varying amino acid lengths, isoelectric points, and molecular weights, and are primarily localized in the nucleus and chloroplast. Chromosomal localization indicated that these genes are distributed across six rice chromosomes. Gene structure analysis revealed diverse exon-intron arrangements, while motif analysis identified 10 conserved motifs. Collinearity analysis showed gene duplication events, suggesting purifying selection. Cis-regulatory element analysis indicated their involvement in abiotic stress responses. The protein–protein interaction analysis revealed that most OsE2F/DP proteins are associated with cell cycle regulation and transcriptional control. Expression analysis revealed tissue-specific expression patterns and differential expression under various stress conditions. Specifically, qRT-PCR validation under cold stress showed that some E2F/DP genes were upregulated at 12 hours and downregulated at 24 hours, indicating their potential role in cold tolerance. These findings provide a foundation for further investigation of the roles of the rice E2F/DP gene family in development and stress responses.

## 1 Introduction

Rice (*Oryza sativa* L.) is one of the world’s most important staple crops, providing food for more than half of the global population. However, rice is highly sensitive to both biotic and abiotic stresses (Ganie et al., 2022; Gao et al., 2007). Among these, abiotic stresses, including excessive or insufficient water, high or low temperatures, and high salinity, are major limiting factors in crop production and can severely affect rice growth and yield (Todaka et al., 2012). Previous studies have shown that cold stress significantly impairs rice growth and development, leading to severe yield losses (Hu et al., 2024).

Abiotic stresses are primary factors negatively affecting plant growth and development. These stresses cause a series of detrimental effects, including changes in plant morphology, physiology, biochemistry, and molecular processes. Specifically, abiotic stresses encompass extreme temperature stress, drought, flooding, salinity, metal toxicity, and nutrient deficiency, with each type inducing distinct responses. Significant progress has been made in understanding these stresses, and several stress-responsive genes have been identified. For instance, the NAC family gene *SNAC1* has been shown to respond to drought, salt, cold stresses, and abscisic acid in rice (ABA), acting as a regulator that mediates stress tolerance through various pathways (Hu et al., 2006). Studies of rice have also demonstrated that the splicing of plant-specific SR proteins can cause functional loss in the *rs33* mutant, which exhibits heightened sensitivity to salt and cold stress (Butt et al., 2022). Moreover, research by Wang et al. has revealed that overexpressing *OsbZIP20* in transgenic rice enhances tolerance to salt and drought stresses and increases sensitivity to ABA (Wang et al., 2022).

In recent years, significant advances have been made in identifying genes related to abiotic stress responses in plants. Among these, the E2F/DP gene family is one of the key families associated with plant abiotic stress and is also involved in regulating the cell cycle, as well as various developmental processes in plants (Yu et al., 2022). Members of the E2F/DP transcription factor family have been studied and extensively identified in several plants, including *Arabidopsis thaliana* (Kosugi and Ohashi, 2002), Moso bamboo (Li et al., 2021), *Zea mays* L (Sánchez-Camargo et al., 2020), *Triticum aestivum* (Yu et al., 2022), *Medicago truncatula* (Ma et al., 2014), and *Solanum lycopersicum* (Divya et al., 2024). In *Arabidopsis*, the E2F/DP family is one of the most representative gene families, comprising three major branches: E2F, DEL, and DP (Perrotta et al., 2021). Research on the E2F branch is well-established, with members such as *AtE2FA*, *AtE2FB*, and *AtE2FC* playing key roles at different stages of the cell cycle, regulating cell proliferation and expression (Rossignol et al., 2002; Abraham and Del Pozo, 2012). In Moso bamboo, studies have shown that *PheE2F/DP* genes are crucial for leaf and root growth, genome integrity, cell viability, and responses to abiotic stresses, with most of the *PheE2F/DP* genes being upregulated under drought and salt stress (Li et al., 2021). In maize, overexpression of *ZmE2F6* promotes root growth, enhancing tolerance to osmotic stress (Cao et al., 2024). In wheat, the E2F/DP gene family is involved not only in drought stress regulation but also in responses to salt and heat stress (Yu et al., 2022). In Medicago, salt stress has been shown to promote the co-expression of E2F and DP genes (Ma et al., 2014). In tomato, the expression of *SlE2F/DP2* and *SlE2F/DP7* is upregulated under heat, salt, cold, and ABA treatments, indicating their involvement in defense gene regulation and transcription factor co-expression, suggesting their key roles in multiple biological processes (Divya et al., 2024). In rice, although previous studies have demonstrated that members of the E2F/DP gene family play important roles in the regulation of the cell cycle (Guo et al., 2007), systematic identification and comprehensive expression analyses of these genes under abiotic stress conditions remain limited.

In this study, we conducted a comprehensive analysis of the phylogenetic relationships, chromosomal locations, gene structures, conserved motifs, gene duplications, and expression patterns of the OsE2F/DP genes in rice. Additionally, we characterized and compared the subcellular localization of these genes, providing strong evidence for their functional properties. The results of this study will be helpful for further analyzing the biological functions of rice E2F/DP family genes. In addition, the findings of this study will contribute to a deeper understanding of the mechanisms by which rice adapts to various abiotic stresses and offer new perspectives for the cloning, characterization, and molecular marker-assisted breeding of rice functional genes.

## 2 Materials and methods

### 2.1 Identification of E2F/DP genes in rice and database screening

The rice genome and GFF annotation file (T2T-NIP (AGIS1.0)) were downloaded from RiceSuperPIRdb (http://ricesuperpir.com/, accessed May 23, 2025). The amino acid sequences of *Arabidopsis* E2F/DP genes were retrieved from the Arabidopsis Information Resource (TAIR) (https://www.arabidopsis.org/, accessed May 23, 2025) and subjected to BLASTp alignment. The Hidden Markov Model (HMM) file corresponding to the E2F/DP domain (E2F_TDP: PF02319) was downloaded from the Pfam protein family database (http://pfam.xfam.org, accessed May 23, 2025). HMMER 3.0 (http://hmmer.org/, accessed May 23, 2025) was used to search the rice genome database for genes containing the E2F domain. The potential E2F/DP genes were further verified through Pfam (http://pfam.xfam.org/, accessed May 23, 2025), SMART (https://smart.embl.de/, accessed May 23, 2025), and InterPro (https://www.ebi.ac.uk/interpro/, accessed May 23, 2025) databases. The identified OsE2F/DP protein sequences underwent a conserved domain search using the NCBI Conserved Domain Database (*https://www.ncbi.nlm.nih.gov/Structure/cdd/wrpsb.cgi?tdsourcetag*, accessed May 23, 2025). Protein sequences missing E2F/DP-related domains, namely those without the E2F_CC-MB, E2F_TDP, DP_DD superfamily, and DP domains, were eliminated from the dataset. Only the longest transcript of each gene was retained for further analysis.

### 2.2 Prediction of physicochemical properties and subcellular localization

The ProtParam tool (*https://web.expasy.org/protparam/*, accessed May 26, 2025) was used to calculate the physicochemical properties of the predicted rice E2F/DP proteins, including amino acid sequence length, theoretical isoelectric point (pI), molecular weight (Mw), instability index, aliphatic index, and hydrophobicity (GRAVY) score.

Subcellular localization of the identified rice E2F/DP proteins was predicted using the WoLF PSORT online tool (*https://wolfpsort.hgc.jp/*, accessed May 26, 2025), and the most probable localization site was selected as the result. The full-length coding sequence of *OsE2F6* was amplified and inserted into the pCAMBIA1301-35S-GFP vector. Subsequent subcellular localization analysis was performed following the method described by Trinidad et al (Trinidad et al., 2021).

### 2.3 Chromosomal localization

To visualize the chromosomal localization of OsE2F/DP genes, we used the Gene Location Visualization module in TBtools v2.225, based on the rice genome annotation files. This allowed for a clear depiction of the distribution of OsE2F/DP genes across the chromosomes.

### 2.4 Phylogenetic analysis, gene structure, and conserved motif analysis of E2F/DP in rice

The amino acid sequences of E2F/DP genes from rice, *Arabidopsis*, tomato, maize, and soybean were used for phylogenetic analysis. Multiple sequence alignment was performed using Clustal, followed by phylogenetic tree construction using the Maximum Likelihood (ML) method in MEGA12.0 software, with a bootstrap value set to 1000. The resulting phylogenetic tree was visualized and beautified using iTOL (*https://itol.embl.de/*, accessed May 29, 2025).

The motif structure of rice E2F/DP family members was analyzed using the online MEME tool (*http://www.omicsclass.com/article/67*, accessed May 29, 2025), with the maximum number of motifs set to 10 and the minimum and maximum motif widths set to 6 and 50, respectively. The gene structures of the rice E2F/DP genes, including CDS and UTR, were extracted from the rice genome annotation files. Finally, visualization was performed using TBtools v2.225.

### 2.5 Collinearity analysis and gene duplication in rice E2F genes

Homologous E2F/DP genes in rice were identified using the BLASTP program, with an e-value threshold set to < e^-10^. The collinearity relationship among rice E2F/DP genes was analyzed using the default parameters of MCScanX. The resulting E2F/DP gene pairs were visualized using TBtools v2.225. To assess selective pressure, the Ka/Ks ratio was calculated using the Simple Ka/Ks Calculator in TBtools v2.225. A Ka/Ks ratio less than 1 indicates purifying selection, which maintains gene function, while a ratio greater than 1 suggests positive selection.

To explore the collinearity between rice and other species, the genomes and annotation files of *Arabidopsis* (TAIR10.55) and *Sorghum bicolor* (v3.1.1) were downloaded from the Phytozome v13 website (*https://phytozome-next.jgi.doe.gov/*, accessed June 1, 2025). Collinearity analysis between rice, *Arabidopsis*, and sorghum was then performed using MCScanX.

### 2.6 Protein-protein interaction of OsE2F/DP

To identify potential protein-protein interactions of OsE2F/DP genes, we constructed a protein interaction network using the STRING database (*https://string-db.org/*, accessed October 20, 2025). The required score parameter was set to 0.4, and the false discovery rate (FDR) strictness was set to 5%.

### 2.7 Molecular docking

Protein–protein complex structures of rice E2F/DP proteins were predicted using AlphaFold (*https://deepmind.google/science/alphafold/*, accessed November 8, 2025). The docking models were selected based on the combined predicted TM-score (pTM) and interface predicted TM-score (ipTM) for structural analysis. The resulting complexes were visualized using PyMOL (*https://pymol.org/*, accessed November 10, 2025) to examine key interacting residues and binding interfaces.

### 2.8 Analysis of cis-acting elements in the promoter regions of rice E2F genes

A 2000 bp region upstream of the start codon (ATG) of each rice E2F/DP gene was considered the promoter sequence. Promoter sequences were extracted using TBtools v2.225, and cis-acting elements were predicted using the PlantCARE online tool (*https://bioinformatics.psb.ugent.be/webtools/plantcare/html/*, accessed June 1, 2025). The cis-regulatory elements associated with stress response, plant growth and development, hormone responses, and light responsiveness were visualized and summarized using the Basic Biosequence View and HeatMap modules in TBtools v2.225.

### 2.9 In silico gene expression of E2F/DP genes

The expression profile of OsE2F/DP genes was obtained from the Rice Genome Annotation Project (*https://rice.uga.edu/index.shtml*, accessed June 2, 2025). Heatmaps of gene expression were generated using the TBtools HeatMap module to illustrate the expression patterns of these genes across different tissues and under various conditions.

### 2.10 Plant material and stress treatment

The japonica cultivar “Nipponbare” was used as the plant material. Seeds were surface sterilized with 75% ethanol and 2% sodium hypochlorite before being planted in autoclaved nutrient soil. The plants were grown in a controlled greenhouse environment, with a temperature of 25°C and a light cycle of 8 hours of light and 16 hours of darkness. At the three-leaf stage, uniform seedlings were transferred to a growth chamber set to 4°C for cold treatment. Leaf samples (the second fully expanded leaf) were collected at 0, 6, 12, and 24 h, immediately frozen in liquid nitrogen, and stored at −80°C for subsequent RNA extraction.

### 2.11 RNA extraction and quantitative real-time PCR analysis

Approximately 150–300 mg of leaf tissue was collected at each stress time point, immediately frozen in liquid nitrogen, and ground into a fine powder. To ensure statistical robustness, the seedlings were divided into three independent groups (e.g., 2 seedlings per group) to serve as biological replicates. Total RNA was extracted from each replicate using an RNA extraction kit (CW Bio, Taizhou, China) according to the manufacturer’s instructions. RNA integrity was evaluated by agarose gel electrophoresis, and RNA concentration and purity were determined using a NanoDrop ND-2000 spectrophotometer (Thermo Scientific, Wilmington, DE, USA).

For first-strand cDNA synthesis, 1 µg of total RNA was used as the template for each reaction to ensure consistency. Reverse transcription was performed using the PrimeScript™ RT reagent Kit with gDNA Eraser (Takara Bio, Dalian, China). Following the manufacturer’s protocol, the genomic DNA removal step was conducted at 42°C for 2 min. The reverse transcription reaction was then carried out at 37°C for 15 min, followed by enzyme inactivation at 85°C for 5 s. The resulting cDNA products were diluted 10-fold with nuclease-free water and used as the template for real-time quantitative PCR (RT-qPCR) analysis.

To investigate the expression patterns of rice E2F/DP genes under abiotic stress conditions, eight genes were analyzed by qRT-PCR. Gene-specific primers were designed using Primer-BLAST on the NCBI website (https://blast.ncbi.nlm.nih.gov/Blast.cgi, accessed on 6 September 2025). qRT-PCR was performed using SYBR™ Green PCR Master Mix (Thermo Fisher Scientific, Waltham, CA, USA). Each reaction was conducted with three technical replicates, and samples collected at 0 h were used as controls. Relative gene expression levels were calculated using the relative quantification (RQ) method. The qRT-PCR reaction system and thermal cycling conditions followed the protocol described by Mo et al (Mo et al., 2021). The Ubiquitin gene was used as the reference gene for relative expression analysis (De Freitas et al., 2019). All primer sequences used in this study are listed in **Supplementary Table S13**.

## 3 Results

### 3.1 Identification of the E2F/DP gene family in rice

**Figure 1:**
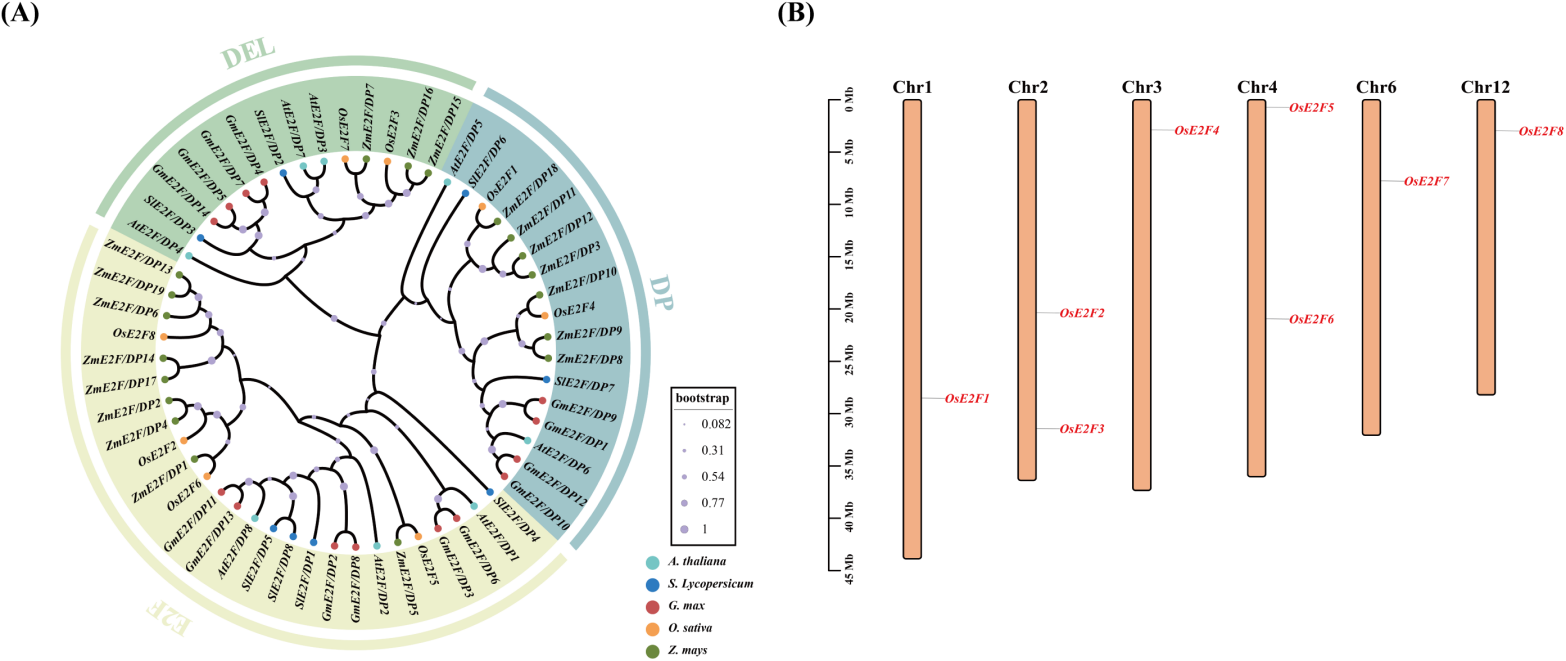
**(A)** Phylogenetic analysis of the E2F/DP gene family in tomato, rice, soybean, maize, and *Arabidopsis thaliana*. The E2F/DP genes were classified into three major groups: E2F-type, DEL-type, and DP-type. **(B)** Chromosomal localization of OsE2F/DP genes. Chromosome numbers are shown at the top, and the scale on the left indicates physical distance in megabases (Mb). A genetic interval of approximately 350 kb was set to accurately illustrate the distribution of the genes.

We identified eight members of the rice E2F/DP gene family, which were named *OsE2F1* to *OsE2F8* based on their chromosomal positions. To investigate the evolutionary relationships between rice E2F/DP proteins and those from *Arabidopsis*, tomato, soybean, and maize, we selected the amino acid sequences of E2F/DP genes from these species for phylogenetic analysis. The analysis included 14 soybean E2F/DP genes, 8 *Arabidopsis* E2F/DP genes, 8 tomato E2F/DP genes, 8 rice E2F/DP genes, and 19 maize E2F/DP genes. The phylogenetic analysis results (**Figure 1A**) revealed that the E2F/DP gene family can be divided into three subfamilies: E2F, DEL, and DP (**Supplementary Table S1**). These subfamilies were unevenly distributed, with the E2F subfamily being the largest, containing 26 members, followed by DP with 17 members, and DEL with 14 members.

### 3.2 Chromosomal distribution of E2F/DP genes in rice

The chromosomal distribution of the E2F/DP gene family in rice has been mapped within the rice genome. The results showed that these genes are located on six out of the twelve chromosomes, although their distribution is uneven (**Figure 1B**). Chromosomes 1, 3, 6, and 12 each contain one OsE2F/DP gene, while chromosomes 2 and 4 each harbor two genes. This widespread chromosomal distribution highlights the evolutionary significance of the OsE2F/DP gene family in rice and suggests their potential functional diversity.

### 3.3 Physicochemical properties of E2F/DP family proteins in rice and subcellular localization

We conducted a detailed analysis of the biophysical and chemical properties of the proteins encoded by the E2F/DP gene family (**Table 1**). The ProtParam tool was used to analyze the physicochemical properties of the OsE2F/DP proteins (**Table 1**). The protein lengths ranged from 231 to 494 amino acids, with molecular weights varying between 26.2 and 50.9 kDa. The isoelectric points (pI) of the proteins ranged from 4.67 to 9.61. Notably, most members had an instability index greater than 40, indicating that they may be relatively unstable *in vitro* according to common thresholds. At the same time, the relatively high aliphatic index (71.2–91.1) suggests that these proteins may have potential for thermal stability. All members exhibited negative GRAVY values (−0.72 to −0.45), indicating that they are generally hydrophilic, which facilitates their soluble expression in the cell. Additionally, we performed subcellular localization prediction for the OsE2F/DP family proteins, and the results showed that these proteins are distributed in both the nucleus and chloroplasts.

**Table 1:** Characteristics of gene structures and protein properties of rice OsE2F/DP family members.

Gene name	Gene ID	Length (aa)	MW (Da)	pI	Instability index	Aliphatic index	GRAVY	Predicted location(s)
*OsE2F1*	AGIS_Os01g041630.mRNA1	294	32859.54	9.61	54.08	81.39	-0.62	Cytoplasmic
*OsE2F2*	AGIS_Os02g029500.mRNA2	475	50980.87	4.95	51.88	75.39	-0.52	Chloroplast
*OsE2F3*	AGIS_Os02g046030.mRNA1	441	48540.11	8.98	45.63	79.89	-0.49	Chloroplast
*OsE2F4*	AGIS_Os03g004600.mRNA1	231	26229.97	5.98	64.51	91.13	-0.45	Chloroplast
*OsE2F5*	AGIS_Os04g001070.mRNA1	494	53766.59	5.03	59.98	71.17	-0.57	Chloroplast
*OsE2F6*	AGIS_Os04g029870.mRNA1	319	35237.98	8.88	62.45	78.62	-0.57	Chloroplast
*OsE2F7*	AGIS_Os06g012270.mRNA1	425	46848.58	8.75	58.97	72.40	-0.72	Nuclear
*OsE2F8*	AGIS_Os12g004970.mRNA2	446	48581.53	4.67	52.82	74.62	-0.53	Cytoplasmic

**Figure 2:**
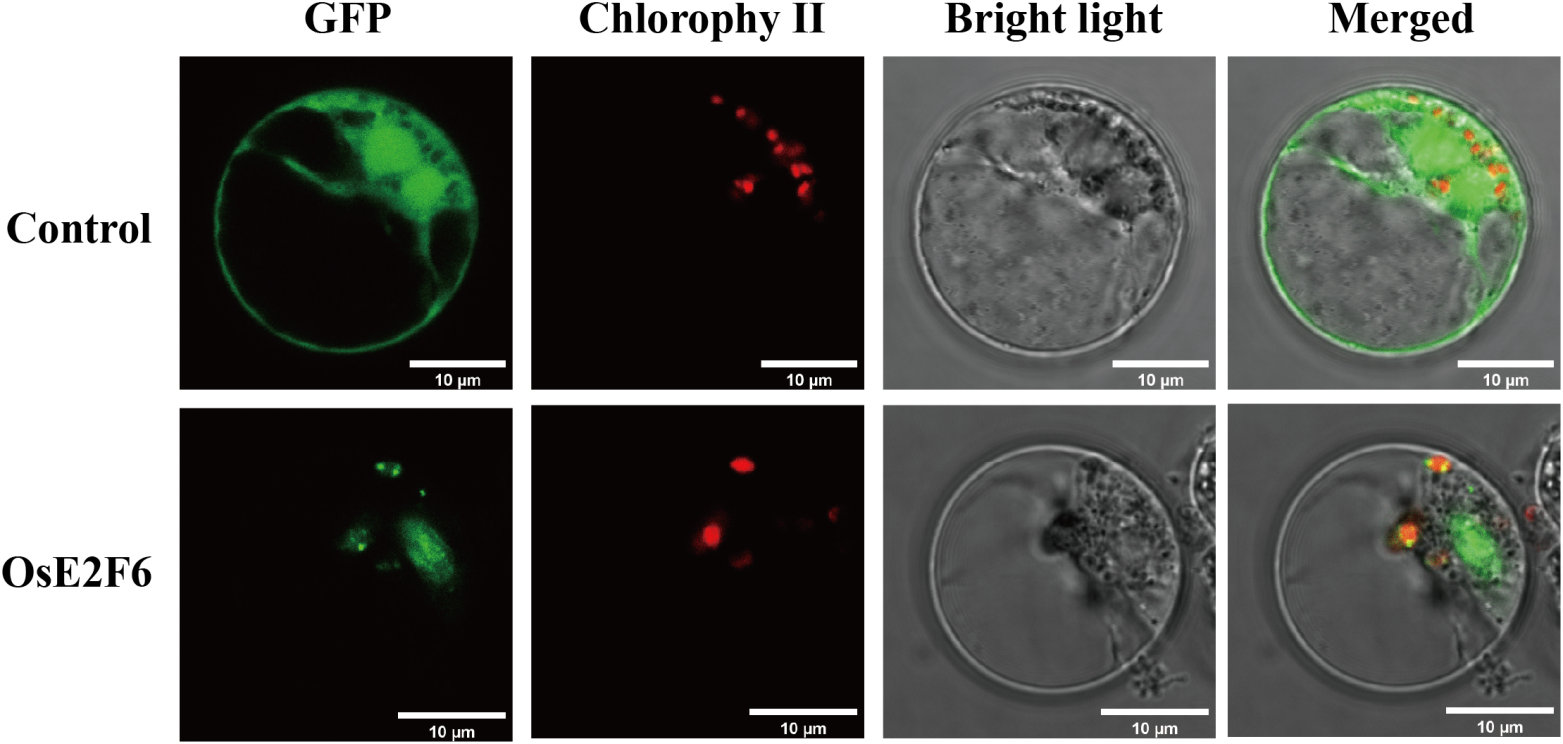
Subcellular localization of OsE2F/DP proteins. Scale bar = 10 μm.

We predicted that the majority of the proteins in the OsE2F/DP gene family are localized to the chloroplasts and nucleus. Based on the expression profiles of rice E2F/DP genes under various stress conditions, we observed that *OsE2F6* exhibited relatively lower expression levels compared to other family members. Therefore, we selected this gene for further subcellular localization analysis. The results showed that the fluorescence signals were primarily localized in the chloroplasts (**Figure 2**).

### 3.4 Gene structure and protein domain analysis of the rice E2F/DP gene family

**Figure 3:**
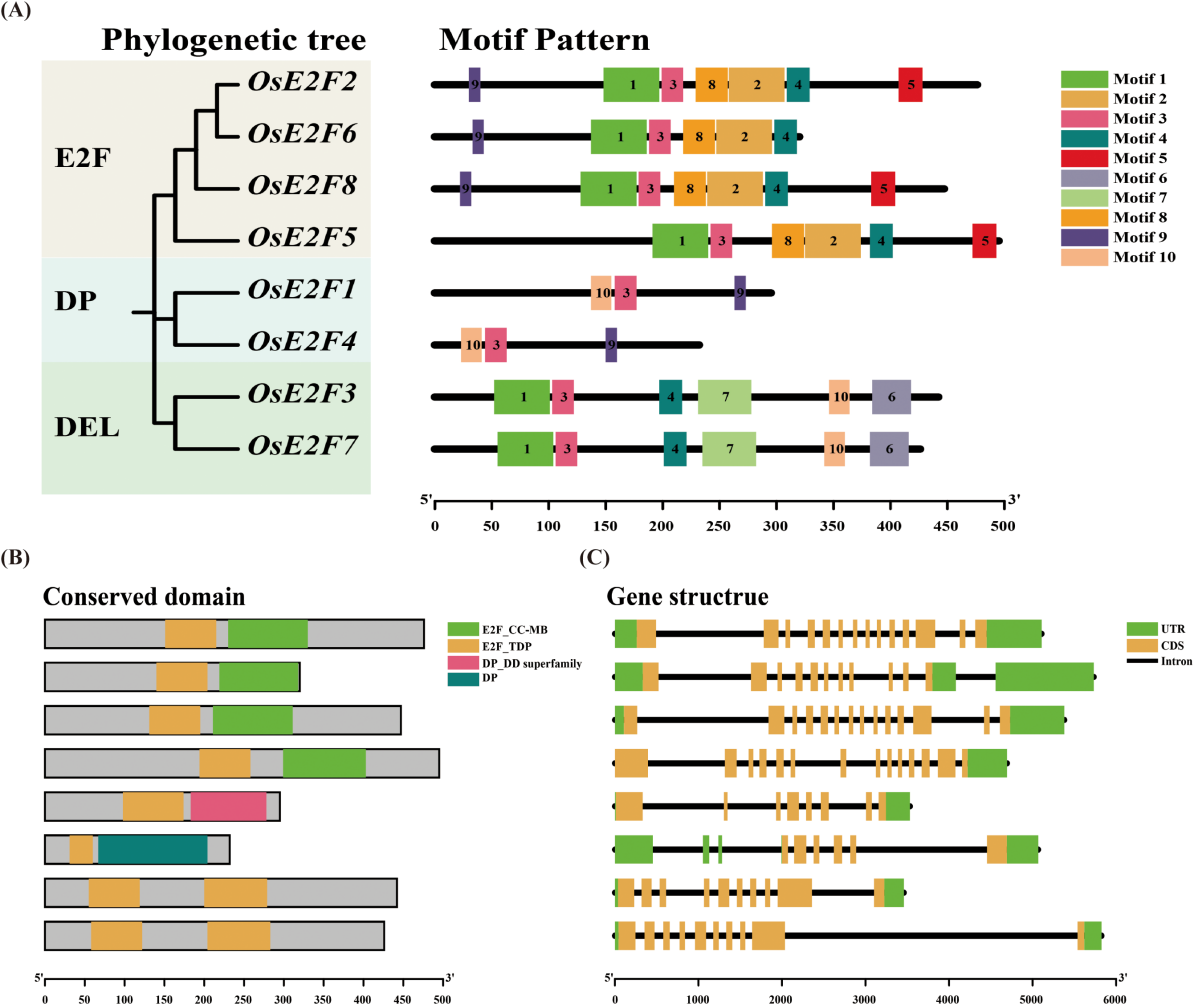
Conserved domains, motif patterns, and gene structures of rice E2F/DP genes. **(A)** The motif patterns of OsE2F/DP members are shown. **(B)** Conserved domains are indicated by colored boxes, with four conserved domains represented by distinct colors. The scale bar at the bottom of the panel allows estimation of the relative lengths of gene structures and motif components. **(C)** The gene structures of OsE2F/DP genes are illustrated, where green boxes represent the untranslated regions (UTRs) at the 5′ and 3′ ends, yellow boxes denote the coding sequences (CDSs), and black lines indicate introns.

To study the homology among rice E2F/DP gene family members, we scanned the amino acid sequences of all OsE2F/DP family members, revealing 10 distinct protein motifs (**Figure 3A**, **Supplementary Table S2**). The number of motifs in each OsE2F/DP gene ranged from 3 to 7. Some motifs exhibited specific patterns based on the family classification, particularly motif 3, which was universally present in all branches, suggesting that this motif may play an important role in the OsE2F/DP family.

Conserved domain analysis was performed to elucidate the function of the E2F/DP family proteins. After sequence analysis, four conserved domains were identified (**Figure 3B**). The results showed that all members of the E2F, DP, and DEL subfamilies possessed the E2F_TDP domain, whereas only the DP subfamily members contained the DP_DD superfamily domain and the DP domain.

The diversity in gene structure supports the functional diversity of the genes. To gain a deeper understanding of the relationship between gene structure, function, and the evolutionary process of OsE2F/DP members, we analyzed the gene structures of the identified OsE2F/DP members. The number of exons in the OsE2F/DP family members ranged from 6 to 14, showing significant variation (**Figure 3C**). Notably, *OsE2F4* had the fewest exons (6), while *OsE2F2*, *OsE2F8*, and *OsE2F5* had the most exons (14). Furthermore, the gene sequence lengths of OsE2F/DP family members ranged from 3000 bp to 6000 bp.

### 3.5 Correlation analysis of rice E2F/DP gene family

**Figure 4:**
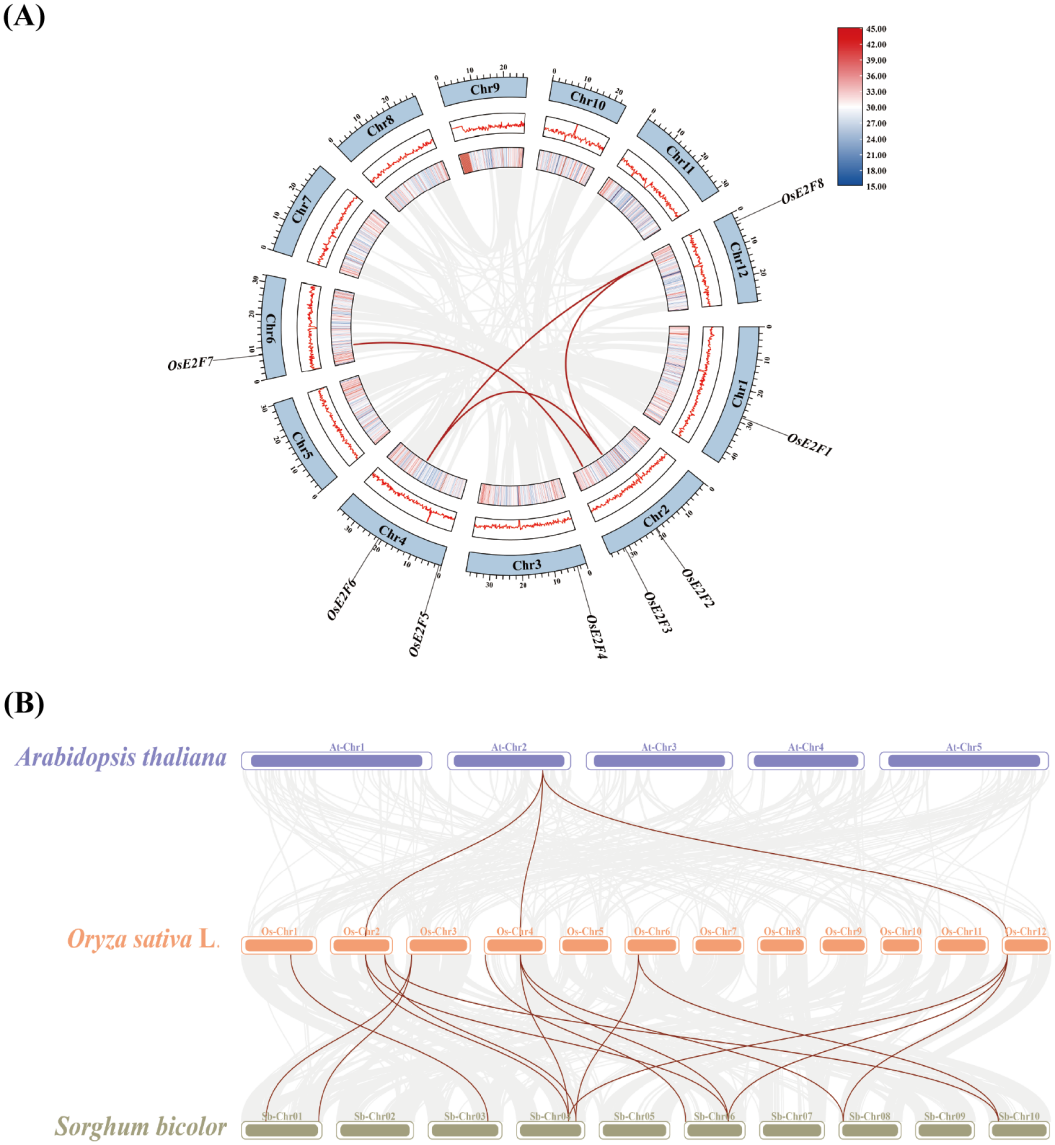
**(A)** Collinearity analysis of homologous OsE2F/DP genes in rice. In the figure, syntenic blocks across the entire rice genome are shown with a gray background, while red curves connect the pairs of homologous OsE2F/DP genes. **(B)** Synteny relationships of E2F/DP genes among *Arabidopsis thaliana*, rice, and sorghum. Gray lines in the background represent collinear gene pairs between the two species, whereas the E2F/DP collinear gene pairs are highlighted with reddish-brown lines.

To elucidate the collinearity between the E2F/DP gene family members, we performed both intraspecific collinearity analysis in rice and interspecific collinearity analysis between rice, *Arabidopsis*, and sorghum (**Figure 4A**). Among the eight identified OsE2F genes, we identified four pairs of duplicate gene pairs, including: *OsE2F2*-*OsE2F8*, *OsE2F6*-*OsE2F8*, *OsE2F2*-*OsE2F6*, and *OsE2F3*-*OsE2F7*. Each pair of duplicate genes was located within the same subfamily, indicating that these genes likely underwent duplication events, but did not fully differentiate, potentially leading to functional redundancy. We also calculated the Ka/Ks ratio of these duplicated gene pairs using TBtools. The Ka/Ks ratios ranged from 0.19 to 0.23, indicating that these gene pairs underwent purifying selection, aiming to eliminate harmful mutations in the species (**Supplementary Table S3**).

Collinearity genes between species are gradually formed during the evolutionary process. To study the origin and evolutionary relationships of the E2F/DP gene family, we explored the homology of the E2F/DP gene family between rice, *Arabidopsis*, and sorghum (**Figure 4B**). The results showed that there were 16 pairs of homologous genes between rice and sorghum, while there were 3 pairs of homologous genes between rice and *Arabidopsis*. The differences in the number of collinearity genes between evolutionary branches suggest that there are differences in the flow and evolution of E2F/DP genes among different species.

### 3.6 Prediction of the E2F/DP family protein interaction network

**Figure 5:**
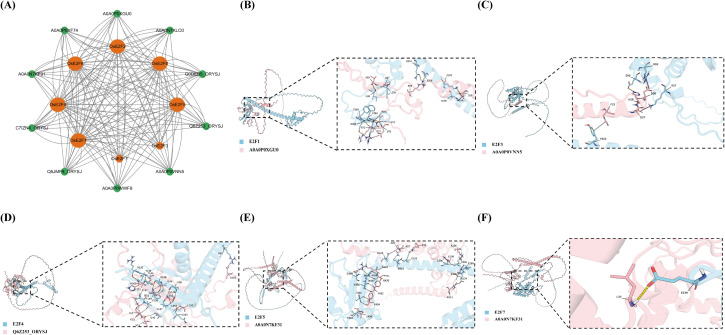
**(A)** Protein–protein interaction (PPI) network of OsE2F/DP proteins. Each node represents a protein, and each edge indicates an interaction between proteins. The orange nodes denote the queried OsE2F/DP proteins. **(B-F)** Predicted structural models and protein–protein docking results of selected OsE2F/DP family members. (ipTM + pTM ≥ 0.5).

To further understand the role of the OsE2F/DP gene family in growth and development, we predicted the protein interaction networks among all OsE2F/DP proteins (**Figure 5A**). Our analysis revealed that most OsE2F/DP proteins are involved in the regulation of the cell cycle and transcriptional control.

Some of these OsE2F/DP proteins interact with proteins involved in recruiting histone deacetylases to control gene transcription, suggesting their important role in plant cell proliferation. Additionally, some OsE2F/DP proteins are predicted to interact with proteins that regulate the G1/S transition. Interestingly, we identified five OsE2F/DP proteins in the predicted interaction network—*OsE2F2*, *OsE2F5*, *OsE2F6*, *OsE2F7*, and *OsE2F8*—that interact with the cDNA cloning protein (Q5ZBT1).

### 3.7 Molecular docking

Based on the results of the protein–protein interaction (PPI) analysis, we performed molecular docking predictions for the interacting protein pairs. Five protein pairs with combined pTM and ipTM scores ≥ 0.5 were selected for further analysis (**Supplementary Table S4**). Specifically, the combined pTM and ipTM scores for the pairs E2F1–A0A0P0XGU0, E2F3–A0A0P0VNN5, E2F4–Q6Z253_ORYSJ, E2F5–A0A0N7KF31, and E2F7–A0A0N7KF31 were 0.57, 0.53, 0.72, 0.51, and 0.50, respectively (**Figures 5B–F**).

Among them, the proteins Q6Z253_ORYSJ and A0A0N7KF31, which interact with E2F4, E2F5, and E2F7, are identified as Tesmin/TSO1-like CXC domain-containing proteins. Proteins containing the CXC domain play a conserved role in regulating organogenesis and cell proliferation. The typical symptom of rice chilling injury is that low temperature will lead to abnormal meiosis of pollen mother cells and pollen sterility, which will eventually lead to the decline of seed setting rate. We speculate that the interaction between E2F/DP gene family and CXC module may alleviate the adverse effects of low temperature stress by recalibrating the developmental plasticity of rice. This interaction provides a potential key mechanism between the cell cycle regulation mechanism and the environmental adaptation of rice reproductive organs.

### 3.8 Cis-regulatory element analysis of OsE2F/DP gene promoters

**Figure 6:**
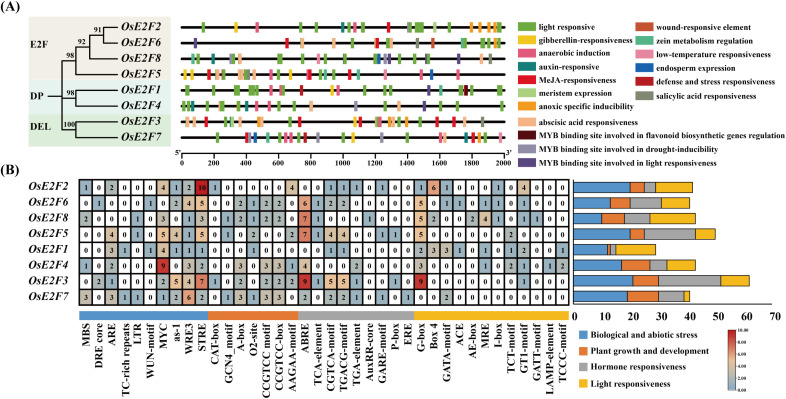
**(A)** The OsE2F/DP gene family promoter sequences were used to predict cis-acting elements. **(B)** The predicted cis-acting elements were organized and visualized.

To explore the potential regulatory functions of the E2F/DP gene family under abiotic and biotic stress, we analyzed their promoter sequences. Ten stress-related cis-regulatory elements were identified in the promoter regions of the E2F/DP genes, including MBS, MYC, ARE, WRE-3, STRE, LTR, DRE core, WUN-motif, TC-rich repeats, and as-1 (**Figure 6B**). Among the eight E2F/DP genes, STRE (salt stress response) and WRE-3 (wound response) elements were present, while MYC (drought response) elements appeared in seven of the E2F/DP genes. ARE (anaerobic response) and as-1 (drought response) elements were present in six genes. MBS (drought induction) was found in four genes, LTR (low temperature response) in three genes, and DRE core (dehydration response), TC-rich repeats (defense and stress), and WUN-motif (wounding response) were found in two genes each. Cis-acting regulatory elements in the promoters of rice E2F/DP genes were visualized using TBtools, and the results showed that the cis-elements in eight OsE2F genes were associated with light responsiveness, gibberellin responsiveness, methyl jasmonate responsiveness, hypoxia induction, and low-temperature responsiveness (**Figure 6A**). These findings suggest that the E2F/DP gene family in rice may be involved in regulating responses to abiotic and biotic stresses, as well as in hormone signaling pathways.

### 3.9 Expression profile of rice E2F/DP genes in various tissues and stress conditions

**Figure 7:**
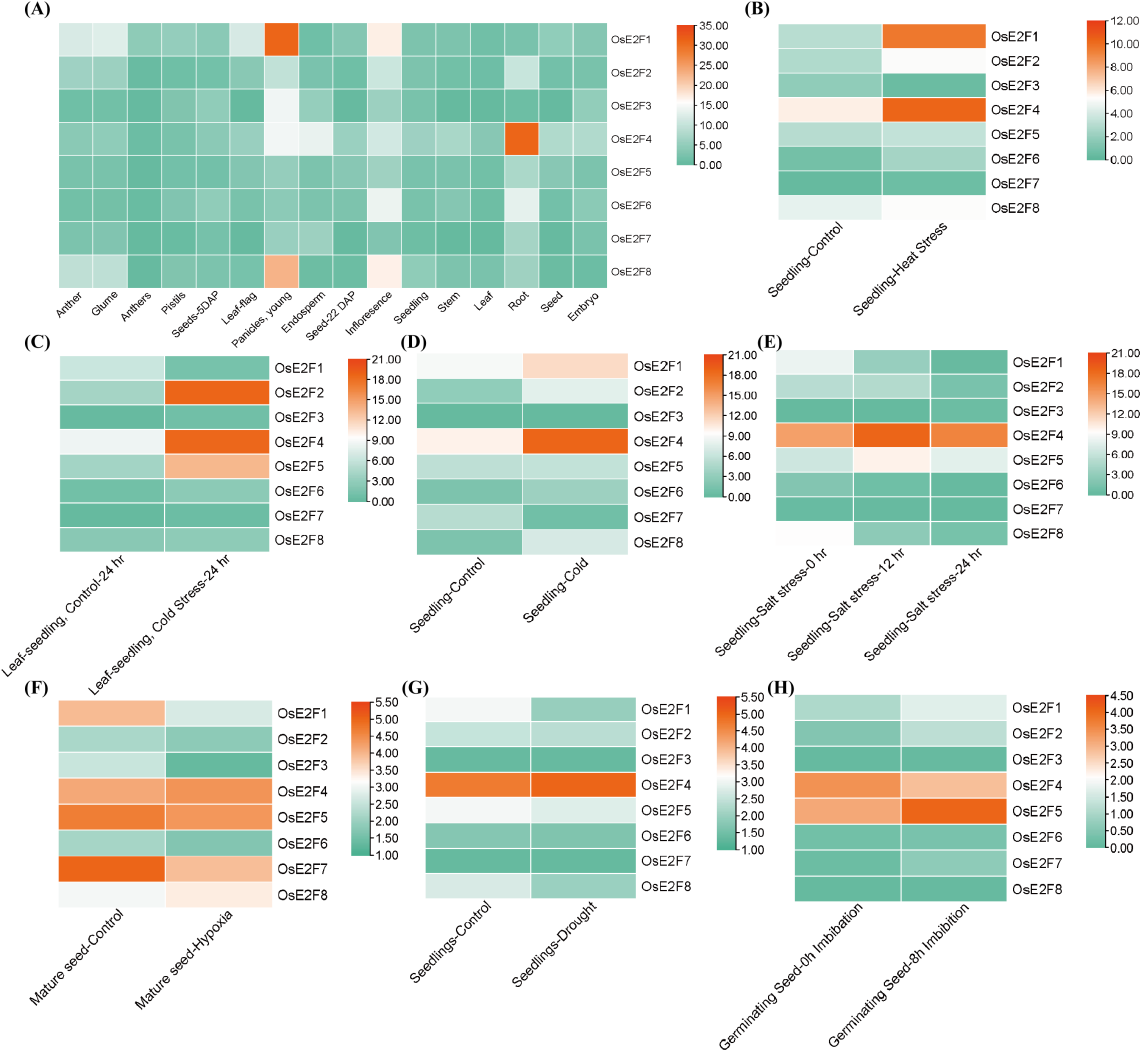
**(A)** Transcriptomic expression profiles of OsE2F/DP gene family members across different tissues. **(B–H)** Expression patterns of OsE2F/DP genes under various stress conditions.

To investigate the expression patterns of the E2F/DP gene family, we examined the dynamic changes of OsE2F/DP genes in different tissues and developmental stages (**Figure 7A**, **Supplementary Table S5**). OsE2F/DP family members are primarily expressed in the roots and inflorescences. Notably, *OsE2F4*, *OsE2F6*, and *OsE2F2* showed higher expression levels in the roots, while *OsE2F1*, *OsE2F8*, *OsE2F3*, and *OsE2F4* were highly expressed in immature inflorescences, and *OsE2F1*, *OsE2F8*, *OsE2F6*, *OsE2F5*, and *OsE2F2* showed higher expression in mature inflorescences. These results suggest that the OsE2F/DP gene family may play a significant role in plant growth and development.

To further study the expression dynamics of OsE2F/DP genes under abiotic stress, we analyzed RNA-seq data for expression patterns under cold stress, salt stress, and combined stress conditions (**Figures 7B-H**, **Supplementary Tables S6**-**S13**). Results showed that *OsE2F4*, *OsE2F1*, *OsE2F2*, *OsE2F8*, and *OsE2F5* were significantly upregulated under low-temperature stress during the seedling stage (**Figure 7D**). *OsE2F2*, *OsE2F4*, and *OsE2F5* showed significant increases in expression in seedlings’ leaves under low-temperature conditions (**Figure 7C**). *OsE2F4* and *OsE2F5* exhibited significantly elevated expression under salt stress at 12 and 24 hours (**Figure 7E**). *OsE2F4*, *OsE2F1*, *OsE2F8*, and *OsE2F2* were significantly upregulated under heat stress (**Figure 7B**), and *OsE2F4* and *OsE2F8* were also significantly upregulated under hypoxic conditions (**Figure 7F**). Notably, *OsE2F4* and *OsE2F5* exhibited higher expression levels under both salt and low-temperature stress.

### 3.10 Expression analysis and qPCR validation of rice E2F/DP genes under cold stress

**Figure 8:**
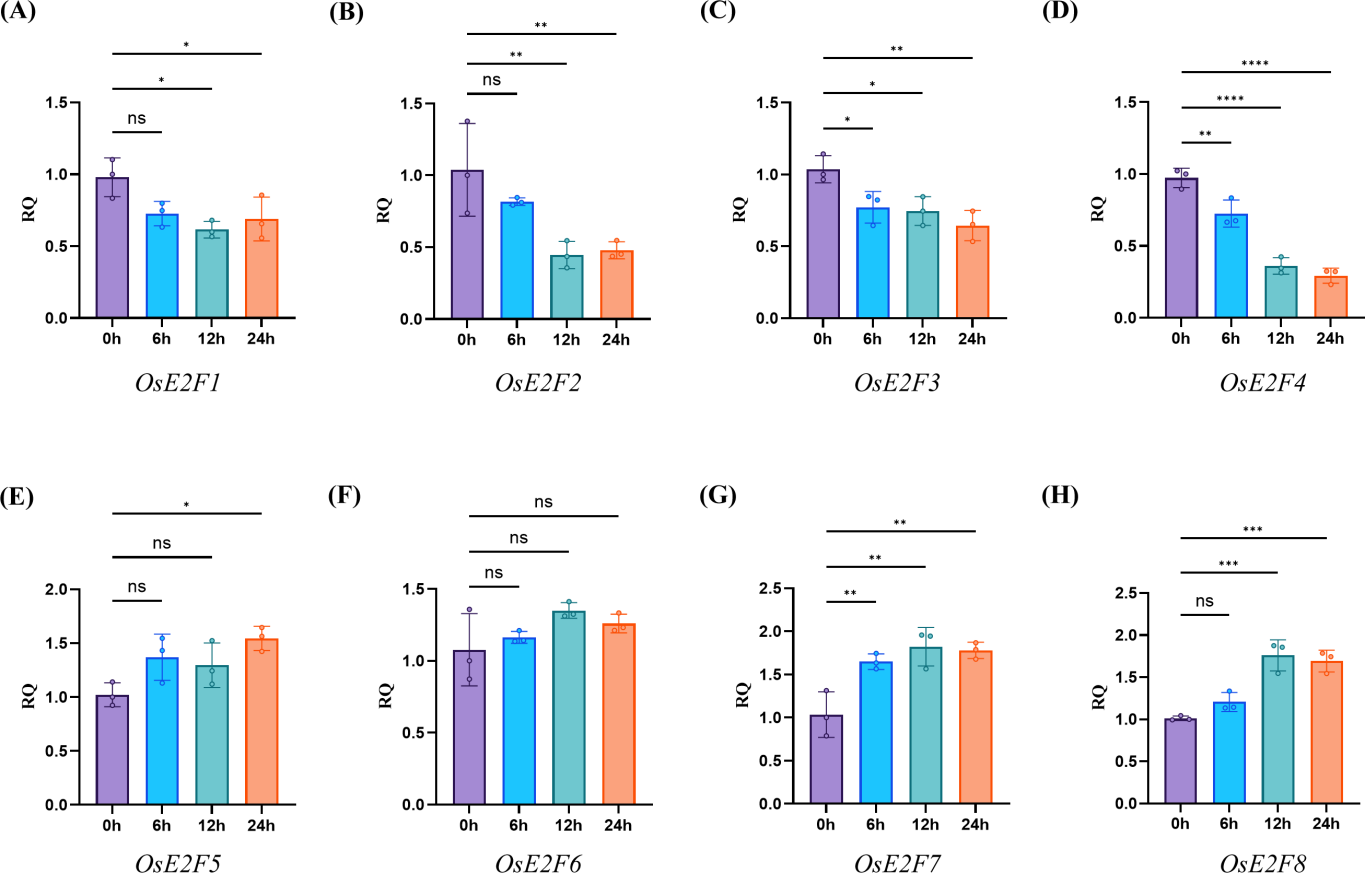
Quantitative RT-PCR analysis of selected representative genes in the rice OsE2F/DP family. **(A)** Quantitative RT-PCR analysis of OsE2F1. **(B)** Quantitative RT-PCR analysis of OsE2F2. **(C)** Quantitative RT-PCR analysis of OsE2F3. **(D)** Quantitative RT-PCR analysis of OsE2F4. **(E)** Quantitative RT-PCR analysis of OsE2F5. **(F)** Quantitative RT-PCR analysis of OsE2F6. **(H)** Quantitative RT-PCR analysis of OsE2F7. **(G)** Quantitative RT-PCR analysis of OsE2F8. Vertical bars indicate the standard deviations. The values represent the mean ± standard deviation (SD) of three independent replicates. An asterisk indicates that the corresponding gene is significantly upregulated or downregulated compared with the 0 h status (*p < 0.05, **p < 0.01, *t*-test).

Based on the expression profile of E2F/DP genes in rice under cold stress, we further analyzed E2F/DP family genes by qRT-PCR after cold treatment for 0, 6, 12 and 24 hours (**Figures 8A-G**). The results showed that *OsE2F7* and *OsE2F8* exhibited a rapid increase in expression at 12 hours, followed by a slight decrease at 24 hours (**Figures 8G, H**). *OsE2F5* showed an increase in expression after 24 hours of cold stress (**Figure 8E**). In contrast, *OsE2F3* and *OsE2F4* displayed a downward trend after 6 hours, with their expression levels significantly reduced at 24 hours (**Figures 8C, D**). Specifically, *OsE2F3* and *OsE2F4* began to decrease markedly at 12 hours, followed by a slight increase at 24 hours (**Figures 8C, D**). The expression patterns of these selected genes were consistent with the transcriptome data. Overall, certain E2F/DP genes in rice respond to cold stress, and the divergent expression trends observed among them may reflect differences in cold tolerance mechanisms in rice.

## 4 Discussion

The E2F/DP gene family has been identified in a variety of plants and mammals (Perrotta et al., 2021). Although the family has been extensively characterized at the genome level in several plants, including tomato, soybean, and wheat, its role in abiotic stress tolerance has been reported in only a few species (Divya et al., 2024). In this study, we utilized the latest rice genome database (Rice Genome Sequencing Project) to conduct a comprehensive characterization of the rice E2F/DP gene family and further explored its role in abiotic stress tolerance, providing essential insights into the mechanisms of rice development and stress responses.

In this study, we systematically identified and characterized eight E2F/DP family genes distributed across six of the twelve rice chromosomes. Previous studies found 27 E2F/DP encoding genes in wheat (Yu et al., 2022), 8 in tomato (Divya et al., 2024), and 7 in soybean (Okay et al., 2023), suggesting that the number of E2F/DP genes is not directly related to genome size or species differences. To elucidate the evolutionary trajectory of the OsE2F/DP gene family in rice, we conducted a comprehensive analysis of their conserved domains and evolutionary features. The E2F_TDP domain was found to be highly conserved across all OsE2F/DP members, underscoring its indispensable role in DNA binding and transcriptional regulation. Such strong conservation suggests that the core regulatory functions of E2F/DP proteins have been stringently maintained throughout evolution. Intragenomic synteny analysis identified four pairs of collinear OsE2F/DP genes, indicating that segmental duplication has been the predominant force driving the expansion of this gene family. Notably, these duplicated gene pairs were primarily distributed within the E2F and DEL subfamilies, implying distinct evolutionary trajectories among different subgroups. Moreover, the duplicated genes exhibited highly similar gene structures, protein lengths, and conserved domain architectures, further supporting their close evolutionary relationships.

E2F transcription factors are well-established activators of genes required for S-phase progression and play central roles in DNA replication and cell cycle regulation (Tyagi et al., 2007; Fouad et al., 2021). During seed germination, reactivation of the cell cycle is a tightly controlled process that operates at multiple regulatory layers, including transcriptional regulation mediated by E2F/DP complexes (Sánchez-Camargo et al., 2020). Previous studies have demonstrated that E2F/DP family members modulate the abundance of cell cycle–related transcripts by binding to E2F/DP cis-elements within target gene promoters (Sánchez-Camargo et al., 2020). Consistent with these observations, our functional annotation analyses revealed that most OsE2F/DP genes are closely associated with cell cycle regulation and transcriptional control, reinforcing their conserved roles in fundamental cellular processes. Importantly, both biotic and abiotic stresses are closely linked to DNA damage, and E2F/DP proteins have been implicated in DNA damage response and repair pathways (Nisa et al., 2023). Under abiotic stress conditions, plants undergo extensive physiological and biochemical reprogramming to rapidly adjust gene expression and adapt to adverse environments (Wani, 2023). These observations raise the possibility that E2F/DP transcription factors may function as integrative nodes linking cell cycle regulation with stress-responsive signaling pathways.

To further explore the potential involvement of OsE2F/DP genes in abiotic stress responses, we performed a comprehensive in silico analysis of cis-regulatory elements within their predicted promoter regions. Numerous stress-responsive cis-elements were identified, suggesting that these genes are transcriptionally responsive to environmental cues. Subsequent analyses of publicly available transcriptome datasets revealed that several OsE2F/DP genes were significantly upregulated in different tissues under cold and salt stress conditions. These expression patterns provide preliminary evidence supporting functional roles of OsE2F/DP genes in abiotic stress adaptation. To validate the transcriptomic results, eight OsE2F/DP genes were selected for qRT-PCR analysis. The results showed that seven of these genes exhibited significant responsiveness to cold stress, corroborating the expression trends observed in RNA-seq datasets. Similar stress-induced expression patterns of E2F/DP genes have been reported in other plant species. In tomato, *SlE2F/DP2* and *SlE2F/DP7* were induced by heat stress and were also markedly upregulated in response to salt stress. Additionally, seven SlE2F/DP genes displayed significant induction at multiple time points during cold treatment (4°C) (Divya et al., 2024). In wheat, *TaE2F1I-19*, *TaDP3III-15*, and *TaDEL2II-27* were significantly induced under cold stress, with *TaDEL2II-27* reaching peak expression within one hour of exposure (Yu et al., 2022).

Collectively, these findings highlight a close association between specific members of the E2F/DP gene family and plant stress responses, suggesting their potential involvement in cold tolerance and broader abiotic stress adaptation. Our results extend the traditional view of E2F/DP proteins as cell cycle regulators by implicating them in stress-responsive transcriptional networks. Nevertheless, the present study is primarily based on bioinformatic analyses. Future studies employing genetic approaches, such as loss- and gain-of-function analyses, together with chromatin immunoprecipitation sequencing (ChIP-seq), will be essential to elucidate the precise regulatory mechanisms by which OsE2F/DP genes mediate stress responses in rice.

## 5 Conclusion

In conclusion, this study provides a comprehensive landscape of the rice E2F/DP gene family, transitioning from genome-wide identification to the elucidation of their pivotal roles in cold-stress adaptation. By integrating phylogenetic analysis, structural motifs, and spatiotemporal expression profiles, we specifically highlight OsE2F2, OsE2F4 and OsE2F5 as the most promising candidates for modulating cold tolerance. Under low temperature stress, these genes showed special expression patterns. Overall, this work provides a foundational framework for functional studies of OsE2F/DP genes and offers promising molecular targets for improving cold tolerance in rice.

## Data Availability

The datasets presented in this study can be found in online repositories. The names of the repository/repositories and accession number(s) can be found in the article/**Supplementary Material**.
